# Dentistry in France

**Published:** 1885-07

**Authors:** K. Holms


### DENTISTRY IN FRANCE.

BY K. HOLMS.

Having noticed in the April number of "Items of Interest" an article by Dr. Guilford on "Dentistry in France," I think it would be unjust, for one who knows something of the subject and who has met with much kindness in France, to allow such misstatements to remain uncorrected.

Dr. Guilford states that, as far as he can learn, "England has but one dental college, and the rest of Europe *none*." He also asserts that "France has not even yet a regularly organized dental society." So far from this being the case, there was a dental college already in operation in Paris in 1880. This was the work of a number of skillful and conscientious dentists, not many of them medical graduates, it is true, but men who had the interests of their profession at heart, and who gave their time and best efforts to the work. This college is known as "*l'Ecole et Hôpital Dentaire Libre*," and is in the Rue Richer.

This, however, did not satisfy the medical men practicing dentistry, so another school was organized and opened in January, 1884, under the auspices of the "Odontological Society of France," and under the name of the "*Institut Odontotechnique de France.*" The directors have leased an old palace in the Rue de l'Abbaye, and in the same building are held the monthly meetings of the Odontological Society. This society, founded in 1878, with Dr. Andrieu as president, is the outgrowth of the *Union des Chambres Syndicales*, which dates back several years farther. Dentistry in France may have greatly changed for the better since Dr. Guilford made his short stay in Paris. Certain it is, that to-day the more advanced in the profession visit one another's operating rooms and laboratories, and a man's standing depends entirely upon his skill, and not upon his nationality.

Dr. Guilford's remark, that "the French dentists were very deficient in operative dentistry," would lead one to conclude that he was not fortunate enough to meet the leading men in the profession in France. As a slight evidence of their knowledge of operative dentistry, I have taken the liberty to translate two lectures, given before the students in the new dental school. Are there many Americans who could handle the subjects with more care and accuracy?

The French can hardly be said to be "devoted to their professional advisers," when they are simply devoted to a house or locality, as Dr. Guilford innocently acknowledges farther on in the same paragraph.

As for the French as patients, those who "sit patiently" and "bear pain bravely" are the exceptions, and not the rule. On the contrary, until one has put before them the necessity for their co-operation in the work, as plainly as one would put it before an average American child, they are far more apt to be impatient, restive and troublesome.

---

### DENTAL SCHOOL OF FRANCE.

Lecture upon the Resection of Teeth, by Dr. Andrieu, Clinical Professor at the School.

Dental resection is an operation which consists in cutting away some portion of a tooth. Whether it be cut off even with its neck for the adaptation of an artificial tooth, or only a part of the cutting edge of the crown is taken away, whether the irregularities are to be filed away, or the roughness of one of its surfaces smoothed, a cavity to be excavated to prepare it for filling, or the end of a root to be cut off, when it has been extracted, with the idea of re-implantation, it is always a resection.

Resection is practiced in a great number of cases, of which the following are the principal ones:

1st—Dental Erosion.

2d—Fracture of the Crown.

3d—Application of Pivot Teeth.

4th—Treatment of Dental Caries, preparatory to filling.

5th—Re-implantation.

DENTAL EROSION.—When the erosion is found upon the front teeth, or upon the grinding surface of the molars, if the roughness resulting from it is sufficient to be annoying, or to cause inflammation to the tongue, or even to give a disagreeable appearance to the teeth, when it is upon the labial surface of the crown, if it shows itself by a groove which is not deep enough to require filling, but which is so marked that the deposits of food in it would cause decay, then certain rules are necessary, which I shall give you later.

Fracture of the Crown.—A person, in consequence of some external violence, a fall, a blow, or biting upon some hard substance in the food, breaks the crown of one or several teeth.

This accident must be remedied. Two conditions present themselves: either the crown is injured to a considerable extent, too large a portion being lacking to think of preserving the tooth, yet not enough to make it necessary to extract the root for the purpose of inserting an artificial tooth, or the crown being only chipped, the deformity produced by the injury is so slight that it is proper to preserve the part that remains in the best condition possible.

In the first of these cases, it is proper to resect the whole crown to the level of the neck, and to fit the root to the artificial tooth to be applied; in the second, it is necessary simply to cut away the unevenness, the sharp angles upon the edge of the fracture which would only break anew, and finally to smooth the surface of the resection, so that neither the tongue, lips, nor cheeks may be irritated.

This is the general rule. But in the partial fracture of the crown there are two conditions to notice: either the pulp has been injured, or it has not.

If it has been, it is necessary, before going on with the resection and in order to avoid pain, to commence by extracting the pulp, then to fill the canal, either provisionally or permanently. If it has not been injured, you must try to avoid pulpitis by all the therapeutic means to which recourse is had in such cases, and never to go on with the resection until the dentine has become nearly insensible.

And at this point I call your attention to an important fact which has a bearing upon partial fractures of the crown, where the pulp chamber is not penetrated, as well as upon teeth attacked by erosion. This is, that the age of the patient has much to do with the success of the operation. It is imprudent to practice resection as long as the pulp chamber is very large in proportion to the size of the crown, and it is absolutely necessary to wait until the dentine becomes thick enough to shrink this cavity, and to protect the pulp against any risk of inflammation. I regard it as a good rule never to undertake this operation until the patient has reached the age of eighteen or twenty years.

I shall return to this subject, *à propos* of resection, as permanent treatment of superficial decay. I shall content myself with telling you that slight cauterization, several times repeated, of the most sensitive parts of the dentine in question, tends to the production of the ivory around the pulp chamber.

Artificial Teeth.—When it is a question of putting in artificial teeth, on pivots, with bands or with caps, with or without clasps or springs, upon natural roots, it is necessary to shape the ends of these roots so as to admit of a perfect adaptation of the tooth.

The surface of the root should be clean, and free from any irregularities in which food might lodge; it should be even with or a little above the gingival margin, so that upon the labial face of the gums the artificial teeth may crowd the tissues sufficiently to appear to grow out of the gums as the natural teeth do, and so that upon the lingual side the plate or cap, if there be one, may be so hermetically applied that the adherence is perfect.

The most important point, indeed, when you undertake to make use of roots as supports for artificial teeth, is to make sure that food cannot penetrate between the roots and the teeth or plates and remain there.

It is unnecessary to tell you that when pivot teeth are in question, it is proper to fill the canals of the roots thus resected, for without this precaution these canals become the receptacles for all sorts of foreign matter, more or less septic, followed by a rapid deterioration of the dentine, with its train of annoyances, such as bad odor, purulent oozing, inflammation of the gingival margin, alveolo-dental periostitis, and sooner or later the entire loss of the roots.

---

Lecture given the 12th of December, 1884.

Dental Caries.—We have recourse to resection in the treatment of dental caries, either as a preparatory step before filling and for the purpose of facilitating access to the cavity, or as treatment pure and simple of superficial caries. As a preparation for filling, resection is practiced:

1st. In order to obtain access to the cavity and to facilitate the various steps in the filling.

2d. To remove all the deteriorated portion, and to shape the cavity so as to hold the filling securely.

Resection, as a means of giving access to the cavity, has given rise to numerous discussions, and all dentists do not agree upon the advisability of having recourse to it when the decay takes place upon the proximal surfaces of the teeth, particularly of the bicuspids or molars. There are, indeed, two theories upon this very interesting point in the practice of dentistry.

Some dentists pretend that resection is useless in obtaining access to the carious cavity, because one can always separate the teeth sufficiently with cotton, rubber or wood, or with various metallic appliances used for this purpose, such as the Jarvis screw, the Bogue separator, etc.

That it is always imperative to restore the injured portion by a filling which will give to the organ its original form, and in extreme cases to reconstruct nearly the whole tooth.

That it is the only proper means of preventing food from crowding between the teeth, from lodging there, and from becoming the cause of decay; in one word, to give back to the teeth their functions in their integrity. They hold that the enamel, being the principal protecting tissue of the tooth, should be respected as much as possible when it is intact, as resection is inadvisable when it takes away more than is necessary.

Finally, that a resected tooth is a deformity, and cannot be pleasing to the eye. Others maintain that after a certain age the temporary separation of the teeth is very painful, and in some instances inevitably results in alveolo-dental periostitis. That it is impossible to properly clean the interstices in which the fillings are in contact, and consequently impossible to prevent the circumference of the fillings thus made from deteriorating sooner or later, particularly at the necks of the teeth, under the margins of the gums. That the parts of the fillings that jut out beyond their attaching points cannot long resist the efforts of mastication, and very soon give way. That in order to fill properly upon the proximal surfaces, it is necessary to prepare a way sufficient to reach the carious cavity. That in order to have the filling secure it should not go beyond the borders of the cavity which it fills. That the only rational means of preventing the ulterior attacks of decay upon the circumference of the fillings consists in the frequent and thorough cleansing of the interstices in which the fillings are, and that resection, practiced according to certain rules, of which I shall tell you later, can only be followed by this result. That, finally, a tooth cleanly cut is quite as agreeable to look at as a tooth from which a lump of gold or amalgam juts out.

You see, gentlemen, that the partisans of these methods have very excellent reasons for the support of their systems. My colleague, Dr. Bogue, defended the first of these systems with great talent in a remarkable paper read at the last meeting of our society, supporting it by numerous considerations, anatomical, physiological, and æsthetic. Without being so thoroughly a partisan of this method, which I propose to criticise at the next meeting of the society, I acknowledge that he furnishes many excellent reasons supporting his way of thinking; but I also see, and this is the most serious reproach I have to make to him, that he is far more theoretical than practical. Indeed, to make contour fillings upon the proximal sides of the teeth, it is first necessary to separate the teeth, which takes time, and is sometimes very painful; I may add that this separation being nearly always possible to obtain in the front teeth, I shall confine my observations to the bicuspids and molars.

In these cases you are obliged to build out the tooth with cohesive gold, and you already know, from the fillings that you have seen made with this gold, the large amount of time needed for it. I do not even speak of the inherent difficulties in such restorations when they are large; the use of the rubber dam has obviated many of them, and it is the operator's business to acquire sufficient facility to succeed in them; I only speak of the time required, and the fees which a dentist has a right to demand for such exact work.

I am convinced that, out of ten patients in any ordinary practice, you will scarcely find two who would be willing to give the time and money necessary for such operations. It is useless here to talk about those exceptional practices which permit the dentist to act entirely according to his own judgment, and to exact from the patients fees that are sufficiently remunerative; doubtless they exist, but they are very rare, at least in France, and even in Paris. I have in view, in speaking to you thus, the ordinary mass of patients who regard the care that they are obliged to give to the mouth, not as a luxury (that would be an exaggeration), but as a need which it is necessary for us to satisfy within very narrow limits, both as to time and expense. It is with regard to them, who are the great majority, that we must find out if the restoration of contour is the most rapid, the most efficacious, and the most practical means of preserving the teeth, and whether it is the one that should be generally adopted.

The second method, which has been followed largely in France, and which has been abused to such an extent that whole dentures have been lost through it, has been defended in America by a very remarkable practitioner, Dr. Arthur. It is practiced by many dentists who use soft, non-cohesive gold; I often have recourse to it myself, and from a practical point of view I can see great advantages in it in a large number of cases. It is simply a question of opportunity. This, gentlemen, is my conclusion. If you adopt either of these methods exclusively, you are quite as likely to make failures with one as with the other, but both of them can be made to render great service when used with discrimination. You must judge for yourselves in just what cases each one should be employed. As a general rule, I should say that upon the proximal sides of the molars, when the carious cavity is small, situated far enough from the neck of the tooth, when the walls are strong enough to support the filling securely, when the crowns touch in such a manner that, the filling once made, food cannot crowd in between them, when one can obtain a sufficient temporary separation, either with the Jarvis or Bogue separator, in order to succeed perfectly in filling, then it is better to restore the contour, that is, to restore the original form of the tooth through replacing the lost portion by an equal portion of filling substance.

On the contrary, when the cavity is large, the margins of its orifice thin, chipped and incapable of affording solid anchorage for the filling, and it seems evident that however good the work may be it can be only very short lived, then, in my opinion, there should be no hesitation. It is proper to resect the margins of the cavity considerably, so as to form of that which remains and the filling material a surface like an inclined plane, and so perfectly smoothed as to make it impossible for it to retain food, and so directed as to permit of the easy cleansing of the interstice, which should have the form of a V, with the top toward the gums. This is the rule, gentlemen, which seems to me proper to adopt in order to be sure of good results.

It is, moreover, the one which had the strongest advocacy in the discussions which took place on this subject at the International

Medical Congress in London, and the one which is followed by all practitioners who have no exclusive system, and who have the best interests of their patients at heart. Besides, it is quite evident that there are exceptions to all rules, but you will understand that it is impossible to enumerate them all in one lesson, and that only the wisdom and experience of the operator can indicate to him his line of action.

I now come to the preparation of the cavity itself, the removal of the diseased portions, the making of points of anchorage, and finally, the shaping of the cavity in such fashion as to hold the filling securely. But I do not wish to intrude upon the domain of your professor of operative dentistry, so I shall content myself with telling you, in a general manner, that the decayed part should be, if possible, entirely removed, particularly around the edge of the cavity; that if you are obliged to leave some portion of softened dentine, as it sometimes happens, to avoid touching the pulp, it should only be at the bottom of the cavity, and finally, that the edges of the cavity should be strong enough to make a solid support for the filling.

